# The Institutes of Medicine

**Published:** 1858-09

**Authors:** 


ART. IV.—*The Institutes of Medicine.* By Martyn Paine, A. M., M. D., &c. New York: Harper & Brothers. 1858. pp. 1095.

This able and interesting work was first issued in the year 1847, and "respectfully dedicated to the medical profession of the United States." That it has not met from the profession a reception such as its merits justly deserve, is very evident from the fact that more than ten years elapsed before a fourth edition was called for. It first saw the light at a time when the humoral and chemical doctrines of life were in the ascendancy, and when vitalism was scouted as an obsolete relic of by-gone ages. But now, when the opinion begins very generally to prevail that the physical doctrines of life will not suffice for the satisfactory solution of the varied phenomena of organic being in health and disease, nor for the explanation of the *modus operandi of remedies*, there is evidently a commencing reäction in favor of the doctrines of vitalism; and this, with the "commentaries" of our authors, begin to be sought for with avidity, and perused with an interest strikingly in contrast with the indifference with which they were at first received. This must be greatly gratifying to Prof. Paine, who, with far-reaching foresight, saw very clearly that a system of medical philosophy based on the laws of the inorganic world, could not stand when brought to the test of observation and experiment. On reading the "Institutes," we cannot but be struck with the admirable consistency of the author's views throughout the entire work; the same principles, the same philosophy from the foundation and substratum of the whole. There is no inconsistency, no contradiction, not even the shadow of any clashing throughout. Taking up each topic in its natural order, as each successive one emanates from, or depends upon the preceding, there is a lucid order every where displayed—a chain, with no broken link; as in a mathematical demonstration, each stop prefaces the way, and is necessary for the succeeding—the demonstration proceeds with logical exactness and unbroken sequence, till the conclusion rests on a basis impregnable as truth itself.

As the author truly remarks, this is the first effort that has ever been made to present the natural relations of the whole subject of the institutes of medicine, including physiology, pathology and therapeutics, in their just order—to paint out the affinities, and to exhibit throughout the important laws and essential foundations of vitalism and solidism, and to maintain throughout a consistency of facts and of laws that shall stamp the whole as the *philosophy of medicine;* and this has been most successfully accomplished; and the zeal, learning, and genius, displayed in its accomplishment, will ever stamp the author as a leading spirit in our profession—as one of the great masters in our art. If the work bears something of a controversial aspect, it was unavoidable in carrying out the great design of the writer. A simple expression of facts, of experience, and of philosophical doctrines, would not have sufficed. It was necessary to expose and refute the errors with which the subject was environed. Obstacles were to be removed before the truth could be fairly reached, inasmuch as "the establishment of truth in medical philosophy, can only be effected by a simultaneous refutation of the errors which surround it."

As our object is not an elaborate review of this work, but only a brief notice of the subjects discussed, with special reference to the author's views regarding the operation of remedies, through the reflex function of the nervous system, we pass on to remark that the physiological portion, embracing 400 pages, treats of: 1st. The composition of organic beings; 2d. Their structure; 3d. Their properties; 4th. Their functions; 5th. Modifications of properties and functions, which arise from sex, temperament, climate, habits, age, &c.; 6th. The relations of organic beings to external objects; 7th. Death.

Passing over the chapters devoted to the composition and structure of organs, we find in the "Third Division," embracing the consideration of the properties or powers of life, "the great focal point from which all emerges that is embraced in medicine; the bond which unites every branch of the science." The author takes as his point of departure, the great and important law, that morbific causes, external or internal, determine disease upon the tissues of one compound organ or another, according to the particular instances of the morbific causes, and in accordance, also, with the natural modifications of the vital properties in every part, and the perceptibilities they may acquire from other causes. He demonstrates, also, with great force and clearness, the great fundamental law, that a general coincidence exists between the natural perceptibilities of the properties of life to their ordinary stimuli, and to those of a morbific and of a remedial nature, according to the natural modifications of the vital properties, both in a general sense and in their relation to particular parts. He shows that it is through this law that the natural stimuli of life maintains all parts in their precise conditions —that morbific agents alter those conditions in certain uniform ways, and remedial agents establish certain other changes which enable the properties and actions of every part to return spontaneously to their natural states; thus embracing an immense range of facts in physiology, pathology and therapeutics, and grouping together many fundamental principles.

The particular mechanism through which these results are brought about, is the nervous system; through the medium, chiefly, of reflex influence, or, as more particularly pointed out in a subsequent chapter on counter-irritants, through those "sympathetic processes which take their origin in cerebro-spinal nerves, *along with the sensitive fibres of the sympathetic, and terminate in the motor fibres of the ganglionic system.*" (Chap. 643.) The author next proceeds to demonstrate the existence of a *vital principle*, as the fundamental *cause* of growth, nutrition, and all other phenomena of organic beings, and in connection with this, the *vital properties*, *irritability*, *sensibility*, *mobility*, *vital affinity*, *vivification*, and the *nervous power*, (substituting the latter in connection with sensibility, for *sympathy*, which is regarded as a *function* and not a property.) These properties of life are represented as the fundamental cause of all healthy and morbid phenomena, —liable to be more or less diverted from that natural state by a variety of causes,—these new conditions constituting the most essential part of disease, —and this instability of these properties lying at the foundation of all disease and of therapeutics. Our aim in the administration of remedies, according to our author, is by acting on these morbid conditions of the vital properties, to change them in such a way as to contribute to their restoration to the natural standard. "The recuperative tendency of the properties of life, when they are driven by morbific causes from their healthy state, enables them to recover spontaneously from the artificial conditions which are substituted by remedial agents for the more intensely morbids."

*Irritability* belongs to all tissues, and is the property upon which all vital agents, external and internal, physical and moral, natural, morbific and remedial, produce impressions in organic life, except as sensibility is concerned in the function of sympathy—mobility may be roused by such impressions when motion follows, either by the direct action of the agent, the indirect or reflex action of the nervous power.

Thus, remedial agents may act directly on the irritability of parts to which they are applied, affecting their functions in a direct manner, or they may call into action the nervous power by their action upon sensibility, thus giving rise to reflex action or sympathy. The same holds true in relation to volition, mental emotions, &c.

In addition to the *common and specific sensibility* of authors, Dr. Paine makes a third kind, *sympathetic sensibility*, which, like specific sensibility, belongs to certain parts only of the nervous system, and is the medium through which impressions are transmitted to the cerebro-spinal axis; in the function of sympathy, perception and sensation being rarely attendant phenomena. This sympathetic sensibility is described as appertaining to what are called the sensitive nerves, and the sensitive fibres of compound nerves, which are, in part, the instruments of common sensibility; but he alludes particularly, as an important anatomical fact, to the existence of sensitive fibres in the sympathetic and pneumogastric nerves, which possess in the most exalted degree; the power of transmitting organic impressions to the nervous centres, though nearly destitute of common sensibility. Through this system of sensitive fibres, Dr. Paine maintains that the whole organic department maintains the specific relations of its several parts, and this sympathetic sensibility, like specific sensibility, being variously modified in different parts, becomes adapted to the reception of impressions from agents of particular virtues, (as different medicinal substances,) and their transmission to the cerebro-spinal axis for the production of true sympathy.

Passing by what is said of *mobility, vital affinity and vivification*, we come to the *nervous power*, which is treated in a highly original and masterly manner, opening up the whole philosophy of the operation of the nervous power in producing motion, under all its aspects, in the phenomena of sympathy induced by morbific and remedial agents, or disease, &c. These views, here presented, had already been brought before the world in the author's "*Commentaries*" and his essay on "*The modus operandi of remedial agents*," but they have not attracted that attention to which they are justly entitled, as they lie at the foundation of physiology, pathology and therapeutics, as taught by the author, and cover the whole ground recently claimed by others, a brief exposition will not be out of place.

The nervous power, we are told, appertains to the vital principle and resides exclusively in the nervous systems, giving rise to results in organic as well as animal life, being far more important in the former than in the latter. Perception is not necessary to its operations, nor does it require a continuity of the nerves with the brain for the function of sympathy, especially in organic life. This nervous power is constantly in operation throughout the organic mechanism, maintaining all parts in harmonious action, and is exerted especially through the motor nerves and the motor fibres of compound nerves, which are mainly dependent for nervous power on the brain and spinal cord. This power, there is reason to believe, is implanted in the motor nerves as well as the brain and spinal cord, though less pronounced in the former; which serves to explain some of the phenomena of contiguous sympathy. Like the other properties of life, sensibility, irritability, &c., this nervous power is capable of being acted upon by external and internal causes, both moral and physical, of being *increased*, or *diminished*, or *altered* in kind, according to the nature of the causes. This power acts as a vital agent to irritability, and is liable to artificial modifications from the operation of physical and moral causes upon the nervous system; its influences upon irritability corresponding with the nature of its modifications, being thus rendered a *vital stimulus*, a *vital depressant*, or a *vital alterative*. The nervous power is brought into universal operation in various ways, according to the seat of the exciting cause; it may be excited in a direct manner by irritants, &c., applied to the brain, spinal cord, or motor nerves: or directly by cerebral or spinal disease, the passions, emotions, imaginations, will, intense reflection, &c., and in all cases will be rendered stimulant, depressant, or alterative to the organic properties and functions, and variously energetic according to the nature of the operating cause, and the intensity and suddenness with which it may operate. In blushing, the power is rendered stimulant; by fear, depressant; by grief, anger, hope, &c., alterative. These effects, whether physical or morbific, are often almost instantaneous. The operation of the nervous power is excited through the medium of sympathetic sensibility, and this complex function results in the true function of sympathy. Impressions are made by physical and moral causes, disease, remedies, &c., upon the foregoing varieties of sensibility, and according, also, to its different modifications in different parts, and the nature of the operating causes. These impressions are then communicated to the cerebro-spinal axis, or to other central parts of the nervous system, and there bring into operation, and variously modify the nervous power. The power thus developed, thus influenced, or so modified in kind that it partakes of the nature of the transmitted impressions, which are more or less coincident with the virtues of the remote causes, is then exerted, through the moter system of nerves, upon the organic properties of distant parts, or of the nervous system itself, by which those properties, and their resulting functions and products are variously effected according to the foregoing circumstances. From this fact, it also results, that the modified conditions which are brought about by the nervous power, when the preternatural operation of this power depends upon external causes, whether morbific or remedial, are more or less analogous to those changes in the organic conditions which are wrought in parts by the direct operation of the same causes. It thence follows that there is imparted to the nervous power, by the foregoing means, more or less of the characteristic virtues of the remote causes, *but under the influence of its own nature*, by which the nervous power is substituted for those causes, and thus reaches, with its acquired attributes and their various effects, every part of the organization.

Upon those directions as a basis, the author erects his entire superstructure of the modus operandi of all remedial agents, whether excitants, depressants, or alteratives; whether they act on the *secretory* and excretory function, or any other function of the body. The term "excito-secretory" is not employed, inasmuch as the term is partial, and refers only to a part of the phenomena thus produced, and does not regard the depressing and alterative influences of the reflex action, as set forth in various parts of the work. This function, "excito-secretory," is, hower, repeatedly described, as one of those embraced within the operation of reflex nervous power, or sympathy, and was distinctly pointed out in the author's essay "on the modus operandi of remedial agents," published as an introductory lecture, in 1840. This reflex function is the leading doctrine and the guiding principle through the whole 299 pages devoted to a consideration of the *functions*, as well as the 109 pages of the pathological portion. We have only space for a very few quotations and references:

"When made upon distant parts, the impression is transmitted to the nervous centres through nerves of sensation or the sensitive fibres of compound nerves, and brings the nervous power in those centres into unusual operation; from which this power is reflected through nerves of motion or the motor fibres of compound nerves, upon the irritability of other parts, or the part which sustained the primary impression, and thus gives rise to those various results which are the prominent phenomena in this complex function." (Page 323.)

Among these results, the therapeutical are always mentioned as among the most important, and they are every where and invariably ascribed to this reflex action. The operation of *emetics, cathartics, diaphoretics, diuretics, alteratives* (as mercury, iodine, &c.,) all of which excite secretions, is every where explained on this principle. Indeed as the author does not admit that medicines operate through absorption, there is no other mode, but reflex action, to explain their operation, except in cases where the medicine is brought directly in contact with the tissue affected; but even here, reflex action is supposed to be influential in producing the given result.

No one can read Dr. Paine's Institutes, without being satisfied that "excito-secretory" is every where comprehended in what is set forth as to the general organic influences of the reflex action.

The general doctrine is again and again reiterated in every part of the work, as on page 661, where the author remarks:

"In approaching again the *modus operandi of remedial agents*, I may again repeat the most essential points,—that the vital principle is a real substantive agent, of which the vital properties, irritability, mobility, &c., are elements superadded to organic beings after the creation of their structure; that the nervous power was superadded only to the animal kingdom; that all organic functions are carried on through their instruments of action, by the four vital properties, which are common to all animated beings; that all vital agents, whether stimulant or sedative, whether natural, morbific, or remedial, operate directly upon these properties, when the nervous power is not concerned in developing motion or changes; that all disease consists in a modification of these properties, and a consequent change of function, and is, therefore, only a variation of the nataral states; that the vital property, sensibility, possesses a modification which I have denominated sympathetic sensibility; that the nervous power is a vital agent, and, like other agents, develops motion and induces changes by acting upon the organic property, irritability, and is exclusively the exciting cause of motion in animal life; that this power or property of the vital principle in animals may be called, in a direct manner, into increased or preternatural operation, by direct impressions, physical or moral, upon the nervous centres, or upon the trunks of nerves; that this power is the efficient agent of remote sympathy, is brought into operation by impressions made upon sympathetic sensibility, *which are transmitted by this property of animal life, through sensitive nerves, to the nervous centres, and these develop the nervous power, which is repeated through motor nerves, upon the irritability of such parts as may be determined by the various influences hitherto expounded, and thus become the exciting cause of motion, of morbific or* THERAPEUTICAL CHANGES, &c., *in those parts upon which its impressions are made;* that the nervous power is susceptible of modifications by the causes which bring it into universal operation, whether physical or moral, and thus, perhaps, *under the influence of its own nature*, of the special virtues of each exciting cause, to which principle is due its alterative effects according to the nature of the exciting causes; and, finally, that a common principle is at the foundation of the philosophy, whether the manifestations of the nervous power be displayed in maintaining the concerted action of the healthy organism, or in deranging that action, *or in restoring disordered movements*, or as the power may be concerned in developing motion, voluntary or involuntary, when propagated immediately from the nervous centres, and without, of course, the sensitive nerves."

We give this long quotation, inasmuch as it embodies the general doctrines, and indeed the fundamental principles and entire philosophy of the Institutes, embracing "excito-secretory," "excito-motory," and every other known function of the nervous system. Nor is reflex, "excito-secretory" action only referred to in general terms; it is set forth by specific facts and illustrations through the whole portion of the work devoted to therapeutics, as in the following passage, which, if we mistake not, also takes in the whole philosophy of the subject and all its most important illustrations:

"When vomiting springs from the operation of tartarized antimony, and often from ipecacuanha, it is only one of the consequences, and a minor one, of the peculiar irritation of the gastro-mucous membrane. Other and far more powerful influences are determined, simultaneously, upon the organic properties and actions of distant and diseased parts (perhaps as distant as the most remote extremity,) by the same nervous power that shook the respiratory organs during the act of vomiting. And often, indeed, does it happen, that those influences are propagated with the most profound effect, when the act of vomiting fails of being consummated, and nausea alone shall send with prostrating effect the modified nervous power over the whole system; when we shall see it simultaneously bathing the whole surface with perspiration; pouring the saliva from the mouth; breaking down a tumultuous excitement of the heart and arteries; starting on the instant a torrent of bile, and an equal effusion from the intestinal mucous membrane; and at the next moment, calling up a magnificent play of sympathies for the evacuation of the fluids, after the manner of an active purgative: these very effusions, also, instituting other circles of sympathy, which join in the great work of curative movements," &c.

It is, however, unnecessary to elucidate this subject at any greater length. It will be recollected that these views were brought before the profession by the author as early as 1842, in his essay "*On the modus operandi of Remedies.*" The connection of the nerves with the function of secretion, was fully demonstrated in the experiments of Wilson Philip, more than thirty years ago, and more recently by those of Bernard; but to Dr. Paine belongs the entire credit of applying these experiments and their results to illustrate the functions of the nervous power as a vital agent, profoundly interested, not only as an "excito-secretory" power, and as a modifying cause of all secreted products, nutrition, &c., but in deducing from them a universal agency of the *reflex action* of the nervous system, through the "double nervous arc" in the production and cure of disease: and it is not too much to claim for our author and countryman, that with unsurpassed acumen and ability, he has abundantly established the fact that secretion in animals is conducted by powers implicated in every part, but that it is constantly influenced physiologically, pathologically, and therapeutically, by reflex action of the nervous system. L.

